# Sequence Characteristics and Phylogenetic Analysis of the *Artemisia argyi* Chloroplast Genome

**DOI:** 10.3389/fpls.2022.906725

**Published:** 2022-06-20

**Authors:** Changjie Chen, Yuhuan Miao, Dandan Luo, Jinxin Li, Zixin Wang, Ming Luo, Tingting Zhao, Dahui Liu

**Affiliations:** Key Laboratory of Traditional Chinese Medicine Resources and Chemistry of Hubei Province, Hubei University of Chinese Medicine, Wuhan, China

**Keywords:** *Artemisia argyi*, *Asteraceae*, chloroplast genome, phylogenetic analysis, variety

## Abstract

*Artemisia argyi* Levl. et Van is an important *Asteraceae* species with a high medicinal value. There are abundant *A. argyi* germplasm resources in Asia, especially in China, but the evolutionary relationships of these varieties and the systematic localization of *A. argyi* in the family *Asteraceae* are still unclear. In this study, the chloroplast (cp) genomes of 72 *A. argyi* varieties were systematically analyzed. The 72 varieties originated from 47 regions in China at different longitudes, latitudes and altitudes, and included both wild and cultivated varieties. The *A. argyi* cp genome was found to be ∼151 kb in size and to contain 114 genes, including 82 protein-coding, 28 tRNA, and 4 rRNA genes. The number of short sequence repeats (SSRs) in *A. argyi* cp genomes ranged from 35 to 42, and most of them were mononucleotide A/T repeats. A total of 196 polymorphic sites were detected in the cp genomes of the 72 varieties. Phylogenetic analysis demonstrated that the genetic relationship between *A. argyi* varieties had a weak relationship with their geographical distribution. Furthermore, inverted repeat (IR) boundaries of 10 *Artemisia* species were found to be significantly different. A sequence divergence analysis of *Asteraceae* cp genomes showed that the variable regions were mostly located in single-copy (SC) regions and that the coding regions were more conserved than the non-coding regions. A phylogenetic tree was constructed using 43 protein-coding genes common to 67 *Asteraceae* species. The resulting tree was consistent with the traditional classification system; *Artemisia* species were clustered into one group, and *A. argyi* was shown to be closely related to *Artemisia lactiflora* and *Artemisia montana*. In summary, this study systematically analyzed the cp genome characteristics of *A. argyi* and compared cp genomes of *Asteraceae* species. The results provide valuable information for the definitive identification of *A. argyi* varieties and for the understanding of the evolutionary relationships between *Asteraceae* species.

## Introduction

*Artemisia argyi* Levl. et Van is a perennial herb belonging to the *Artemisia* genus in the family *Asteraceae*. *Artemisia argyi* contains an abundance of active ingredients, such as flavonoids, phenolic acids, and other volatile components, endowing *A. argyi* with a wide range of pharmacological activities (Mei et al., 2016). For example, 1, 8-cineole and borneol have strong antibacterial and anti-inflammatory activities (Mulyaningsih et al., 2010; Martins et al., 2017; Bansod et al., 2021). Eupatilin is commonly used to treat gastritis and peptic ulcers (Lee et al., 2017; Kim et al., 2018). Moxa wool from *A. argyi* leaves is the main combustion material in moxibustion therapy (Cardini and Weixin, 1998). With the development of the health industry, *A. argyi* is widely used in medicine, healthcare, food, and cleaning products (Liu et al., 2018; Cui et al., 2021); therefore, the market demand for *A. argyi* is growing rapidly.

*Artemisia argyi* is widely distributed in eastern Asia, including China, Japan, South Korea, and Mongolia (Lee et al., 2009). *Artemisia argyi* is rich in germplasm resources, with abundant genetic variations occurring in different ecotypes. Our previous studies demonstrate significant differences in agronomic traits, moxa content, and active ingredients among different *A. argyi* varieties (Chen C. J. et al., 2021). In China, “Qichun Qi AI” (growing in Qichun County, Hubei Province), “Tangyin Bei AI” (Tangyin County, Henan Province), “Ningbo Hai AI” (Ningbo City, Zhejiang Province), and “Anguo Qi AI” (Anguo City, Hebei Province) are the four representative varieties with superior quality. The abundant germplasm resources of *A. argyi* also bring great challenges in planting, and varietal complexity often occurs in cultivation, seriously affecting the yield and quality of *A. argyi*.

The chloroplast (cp) is an organelle in plants and algae (Du et al., 2021; Li et al., 2021) that plays a significant role in plant cell function, including in photosynthesis, carbon fixation, and stress response. The cp is a semiautonomous organelle with a relatively independent genome containing its own genetic system for replication, transcription, and translation (Favier et al., 2021). The cp genome is a double-stranded circular DNA molecule with a conserved quadripartite structure, including a large single-copy (LSC) region, a small single-copy (SSC) region, and two copies of inverted repeat (IR) regions (Song et al., 2017; Gao et al., 2018; Wang M. et al., 2021). The LSC and SSC regions are usually separated by two IR regions.

The comparative genomic studies have demonstrated that most of the angiosperm cp genomes are highly conserved in gene order and content (Chen J. et al., 2021), and generally range in length from 120 to 160 kb (Wang Y. et al., 2021). Angiosperm cp genomes usually encode 110–130 genes (Feng et al., 2020). These genes are divided into three functional categories, namely, photosynthesis-related, transcription/translation-related, and biosynthesis-related genes. Some significant genomic changes have occurred throughout the process of evolution, comprising large-scale genome rearrangements, the contraction and expansion of IR regions, and the loss of specific introns. With the rapid development of sequencing technology, many cp genomes have been reported since the assembly of the *Nicotiana tabacum* cp genome (Shinozaki et al., 1986). Furthermore, cp genomes are extensively used in the fields of species identification, phylogeny, and genetic diversity analysis due to their relatively small size, maternal inheritance, conserved structure, and low nucleotide substitution rate (Qiao et al., 2016).

Although the cp genome of *A. argyi* has been reported previously (Kang et al., 2016), the structural characteristics of the *A. argyi* cp genome and the evolutionary relationships between *A. argyi* varieties remain unclear. In this study, 72 wild and cultivated *A. argyi* varieties were collected from 15 domestic provinces and 1 municipality in China. We sequenced and assembled the cp genomes of these 72 varieties and here report 51 different *A. argyi* cp genomes. Furthermore, we systematically analyzed cp genome characteristics of *A. argyi*, including GC content, gene number and order, repeat sequences, and simple sequence repeats (SSRs). The evolutionary relationships between *A. argyi* and other *Asteraceae* plants were also explored based on their cp genomes. These results provide important information for the identification of *A. argyi* varieties and the evolution of *Asteraceae* plants.

## Materials and Methods

### Plant Materials

In this study, 72 *A. argyi* germplasm resources from 47 regions in China were collected and planted in the *A. argyi* Germplasm Resource Garden at Hubei University of Chinese Medicine (Hubei Province, China; 114.264E, 30.451N). Detailed sampling information is shown in Supplementary Table 1. Maps were drawn using Lantuhui v2.8.0.^1^ Fresh leaves were collected from each variety and immediately frozen in liquid nitrogen prior to DNA extraction for sequencing. We also downloaded 67 cp genomes of plants in the family *Asteraceae* from NCBI,^2^ including the published cp genome of *A. argyi* (Supplementary Table 6).

### DNA Sample Preparation and Chloroplast Genome Sequencing

Total genomic DNA was extracted from each sample using a modified cetyltrimethyl ammonium bromide (CTAB) method (Doyle et al., 1987). Agarose gels (1%) and a Qubit 3.0 Fluorometer (Thermo Fisher Scientific, Waltham, MA, United States) were used to evaluate the quality and quantity of the extracted DNA. Genomic DNA samples were fragmented to a size of ∼350 bp by sonication. Index codes were added to each sample and the NEBNext Ultra DNA Library Prep Kit for Illumina (New England Biolabs, Ipswich, MA, United States) was used for the preparation of DNA libraries. Subsequently, DNA fragments were end polished, A-tailed, ligated, and amplified by PCR. PCR products were then purified with the AMPure XP system (Beckman Coulter, Brea, CA, United States) and the DNA concentration was measured using the Qubit 3.0 Fluorometer. Libraries that passed quality inspection were sequenced by Beijing Nuohe Zhiyuan Technology (Beijing) Co., Ltd.^3^ using the Illumina HiSeq Xten-PE150 platform (Illumina, San Diego, CA, United States). The original fluorescence image files were transformed into short reads (raw data). The program fastp (v0.19.7) was used for clean reads by removing the joints and low-quality sequences (Phred quality < 5), and clean reads were recorded in FASTQ format. The raw data of 72 cp genome sequencing have been submitted to NCBI under the accession number PRJNA821831 and the 72 assembled cp genomes have been submitted to the China National Gene Bank Database (https://db.cngb.org/) under accession number CNP0002996.

### Assembly and Annotation of Chloroplast Genomes

We assembled the cp genomes of *A. argyi* varieties using NOVOPlasty v4.2^4^ (Dierckxsens et al., 2017) and selected the *rbcL* gene of *A. argyi* from NCBI (NC030785.1) as a seed sequence; the default settings were used for all other parameters. The Plastid Genome Annotator (PGA) was used to annotate the cp genomes, using *Artemisia annua* Linn (MF623173.1) as the reference. The annotation results were verified by Geneious 8.0.2^5^ (Kearse et al., 2012). The program tRNAscan-SE^6^ (Lowe and Chan, 2016) was used to determine the boundaries of all tRNA genes. A circular map of the *A. argyi* cp genome was drawn using Chloroplot.^7^ Four junctions between IR and SC regions were verified using high-fidelity PCR amplification and Sanger sequencing. The primers are listed in Supplementary Table 4.

### Feature Analysis of the Chloroplast Genome

Relative synonymous codon usage (RSCU) was analyzed using MEGA7. REPuter^8^ was used to detect the size and location of repeat sequences with a minimal length of 19 bp (Kurtz and Schleiermacher, 1999) and an identity of 90%. MISA^9^ was used to identify SSRs (Thiel et al., 2003). Molecular markers for SSR identification are listed in Supplementary Table 2 (Curci et al., 2016). To reveal highly variable regions in cp genomes for the identification of *A. argyi* varieties, we used MAFFT V7.471 (Kazutaka Katoh, Japan) for the alignment (Katoh and Standley, 2013); DnaSP v6 software was then used for a sliding window analysis (Librado and Rozas, 2009) with the window length set to 600 bp and a step size of 200 bp.

### Comparative Analysis of Chloroplast Genomes

To compare the IR boundaries, cp genomes of nine *Artemisia* species downloaded from NCBI were compared with the *A. argyi* cp genome using IRscope^10^ (Amiryousefi et al., 2018). To determine the differences in the cp genomes of 11 *Asteraceae* species, *A. argyi* was used as the reference, and complete cp genome sequence annotations in BED format were converted and uploaded to the online analysis program mVISTA^11^ (Kawabe et al., 2018).

### Phylogenetic Analysis

The complete cp genomes of 72 *A. argyi* varieties were aligned and uploaded to RAxML v8.2.12 (Stamatakis, 2014) to construct a phylogenetic tree using the maximum likelihood (ML) method, and *A. annua* (MF623173.1) was used as the outgroup. For the phylogenetic analysis of *Asteraceae* species, cp genomes of 67 *Asteraceae* species were selected, and *Platycodon grandiflorus* (Jacq.) A. DC. (MZ 202358.1) and *Codonopsis minima* Nakai. (NC036311.1) were used as the outgroup. The cp genomes of 67 selected *Asteraceae* species were downloaded from NCBI, manually annotated, and aligned using MAFFT v7.471 software. Phylogenetic analyses were performed using the coding DNA sequences (CDSs), LSCs, SSCs, IRs, introns, protein-coding genes common to all of the selected species, and the full-length cp genomes. RAxML v8.2.12 was used to construct the phylogenetic tree with 1,000 bootstrap replicates to estimate node support, and the best tree based on bootstrap scores was used for further analysis.

## Results

### Characteristics of the *Artemisia argyi* Chloroplast Genome

**FIGURE 1:**
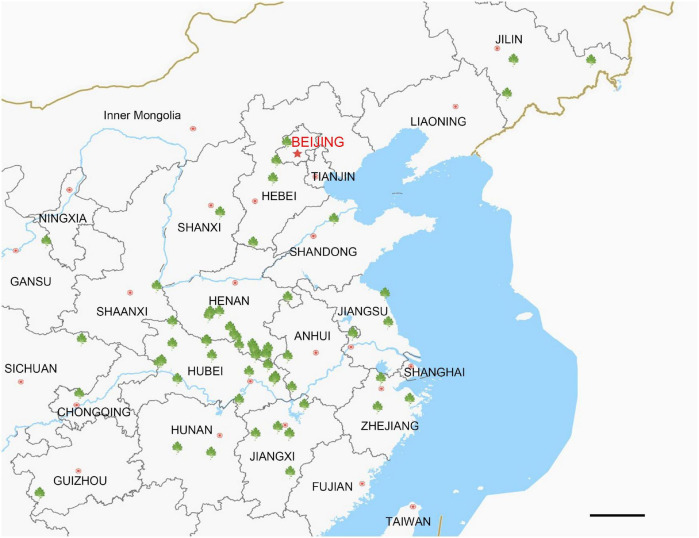
Collection locations for *Artemisia argyi* germplasm resources. The green symbols indicate collection points for *Artemisia argyi* varieties. There are 47 markers located in 15 domestic provinces in China. Scale bar = 20 km.

**FIGURE 2:**
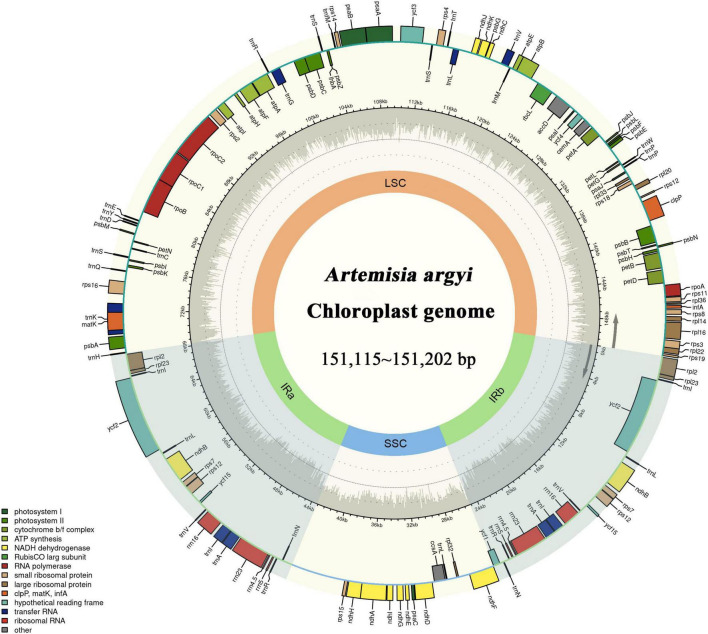
Gene map of the *Artemisia argyi* chloroplast (cp) genome. Genes drawn inside the circle are transcribed in the clockwise direction, and those on the outside are transcribed in the counterclockwise direction. The genes are color-coded based on their function. In the inner circle, darker gray corresponds to GC content and lighter gray corresponds to AT content of the *Artemisia argyi* cp genome. The SSC and LSC regions are separated by two darker areas representing inverted repeat (IR) regions (IRa and IRb).

The locations at which the 72 *A. argyi* varieties were collected are shown in Figure 1. We obtained 32.93–44.18 Mb raw reads for each variety, providing 218- to 292-fold coverage of the *A. argyi* cp genome. Assembly and annotation yielded the complete cp genomes of *A. argyi* varieties. The results showed that the *A. argyi* cp genome was a circular double-stranded molecule ranging in size from 151,115 to 151,202 bp (Figure 2). The *A. argyi* cp genome was composed of the LSC (82,862–82,935 bp), the SSC (18,333–18,371 bp), IRa (24,959–24,968 bp), and IRb (24,959–24,968 bp). The average GC content was 35.6%. Specifically, the LSC, SSC, IRa, and IRb had 35.6, 30.8, 43.1, and 43.1% GC content, respectively. The CDS lengths ranged from 71,805 to 78,654 bp, and GC content in the CDSs ranged from 37.7 to 38.3% (Table 1). On the whole, the structural characteristics of cp genomes were identical between the 72 *A. argyi* varieties, indicating that cp genome structure was highly conserved in this species.

**TABLE 1:** Base composition of *Artemisia argyi* cp genomes.

Region	T (%)	C (%)	A (%)	G (%)	GC (%)	Length (bp)
LSC	32.4	17.5	32	18.1	35.60	82862∼82935
SSC	34.1	16.1	35	14.7	30.80	18333∼18371
IRa	28.3	22.3	28.6	20.8	43.10	24959∼24968
IRb	28.6	20.8	28.3	22.3	43.10	24959∼24968
Total	31.3	18.7	31.2	18.8	37.50	151115∼151202
CDS	31.7∼31.8	17.8∼18.0	29.9∼30.5	20.0∼20.3	37.7∼38.3	71805 ∼78654
CDS-1st	24.7∼24.8	18.5∼18.8	30.3∼31.0	25.6∼26.5	44.3∼45.0	23935∼26218
CDS-2nd	32.4∼32.7	20.4∼19.9	28.6∼29.5	18.0∼18.4	37.9∼38.7	23935∼26218
CDS-3rd	37.9∼38.5	14.1∼15.0	30.6∼31.5	15.9∼16.5	30.0∼31.5	23935∼26218

The *A. argyi* cp genome contained 114 genes: 82 protein-coding, 28 tRNA, and 4 rRNA genes. These genes were divided into three functional categories; there were 47 photosynthesis-related genes, 28 related to self-replication, and 6 genes related to biosynthesis (Table 2). In the IR regions, 18 genes were duplicated in total. Among these duplicated genes, seven were protein-coding (*ndhB, rpl2, rpl23, rps7, RPS12, ycf2*, and *ycf15*), seven were tRNA (*tRNA-UGC, trnI-CAU, trnI-GAU, trnL-CAA, trnN-GUU, trnR-ACG*, and *trnV-GAC*), and four were mRNA genes (*rrn4.5s, rrn5s, rrn16s*, and *rrn23s*). The only single-copy gene located in the IR region was *ycf1*. The results of gene structure analysis showed that there were 13 intron-containing genes in the *A. argyi* cp genome, 12 of which were located in the LSC and one of which was in the SSC (*ndhA*). Of those 13 genes, 11 contained one intron (*atpF, petB, rpl16, trnV-UAC, rpoC1, ndhA, trnL-UAA, trnK-UUU, trnG-UCC, rps16*, and *petD*) and two contained two introns (*clpP* and *ycf3*) (Table 3). We then performed RSCU analysis of the protein-coding genes. The results showed that there were 30 codons with RSCU > 1.00. Notably, there were 29 codons ending with A or U, indicating that the *A. argyi* cp genome preferentially contained synonymous codons ending with A or U (Table 4).

**TABLE 2:** Gene contents in the *Artemisia argyi* cp genome.

Gene category	Gene group	Gene name	Gene number
Photosynthesis	Subunits of photosystem I	*psaA*, *psaB*, *psaC*, *psaI*, *psaJ*, ^b^*ycf3*, *ycf4*	7
	Submits of photosystem II	*psbA*, *psbB*, *psbC*, *psbD*, *psbE*, *psbF*, *psbG*, *psbH*, *psbI*, *psbJ*, *psbK*, *psbL*, *psbM*, *psbN*, *psbT*, *psbZ*	16
	Subunits of ATP synthase	*atpA*, *atpB*, *atpE*, ^a^*atpF*, *atpH*, *atpI*	6
	Subunits of cytochrome b/f complex	*petA*, ^a^*petB*, ^a^*petD*, *petG*, *petL*, *petN*	6
	Subunits of NADH dehydrogenase	^a^*ndhA*, *ndhB, *ndhC*, *ndhD*, *ndhE*, *ndhF*, *ndhG*, *ndhH*, *ndhI*, *ndhJ*, *ndhK*	11
	Large subunit of rubisco	*rbcL*	1
Self-replication	Subunits of RNA polymerase	*rpoA*, *rpoB*, ^a^*rpoC1*, *rpoC2*	4
	Large ribosomal subunit	*rpl12*, *rpl14*, ^a^*rpl16*, *rpl2, *rpl20*, *rpl22*, *rpl23, *rpl32*, *rpl33*, *rpl36*	10
	Small ribosomal subunit	*rps2*, *rps3*, *rps4*, *rps7, *rps8*, *rps11*, *rps12, *rps14*, *rps15*, ^a^*rps16*, *rps18*, *rps19*	12
	Ribosomal RNAs	*rrn4.5S*, *rrn5S*, *rrn16S*, *rrn23S*	4
	Transfer RNAs	*trnA-UGC, trnC-GCA, trnD-GUC, trnE-UUC, ^a^*trnG-UCC*, *trnH-GUG*, *trnI-CAU,*trnI-GAU,^a^*trnK-UUU*,*trnL-CAA,^a^*trnL-UAA, trnL-UAG, trnM-CAU*, * *trnN-GUU, trnP-GGG, trnP-UGG, trnQ-UUG*,*trnR-ACG, trnR-UCU, trnS-GCU, trnS-GGA, trnS-UGA, trnT-UGU,*trnV-GAC,^a^*trnV-UAC, trnW-CCA, trnY-GUA, trnfM-CAU*	28
Biosynthesis	Translational initiation factor	*infA*	1
	c-type cytochrome synthesis gene	*ccsa*	1
	Envelope membrane protein	*cemA*	1
	Subunit of Acetyl-CoA-carboxylase	*accD*	1
	Protease	^b^ *clpP*	1
	Maturase	*matK*	1
Unknown function	Conserved open reading frames	*ycf1*, *ycf2*, *ycf15*	3

*Genes with two copies. ^a^Genes have one intron. ^b^Genes have two introns.

**TABLE 3:** The length of exon and intron in intron-containing genes of the *Artemisia argyi* cp genome.

Gene	Location	Size (bp)	ExonI (bp)	IntronI (bp)	ExonII (bp)	IntronII (bp)	ExonIII (bp)
*atpF*	LSC	1254	145	699	410		
*petB*	LSC	1394	6	746	642		
*rpl16*	LSC	1427	9	1019	399		
*trnV-UAC*	LSC	648	38	573	37		
*clpP*	LSC	1808	69	799	291	610	39
*rpoC1*	LSC	2823	450	732	1641		
*ndhA*	SSC	2169	552	1077	540		
*trnL-UAA*	LSC	505	37	418	50		
*trnK-UUU*	LSC	2640	37	2568	35		
*ycf3*	LSC	1950	126	703	228	740	153
*trnG-UCC*	LSC	799	47	729	23		
*rps16*	LSC	1101	40	864	197		
*petD*	LSC	1158	8	675	475		

**TABLE 4:** Codon usage and codon-anticodon recognition patterns of the *Artemisia argyi* cp genome.

Amino acid	Codon	Number	RSCU (%)	Amino acid	Codon	Number	RSCU (%)
Phe (F)	UUU	954	1.27	Tyr (Y)	UAU	765	1.53
Phe (F)	UUC	552	0.73	Tyr (Y)	UAC	233	0.47
Leu (L)	UUA	779	1.64	Stop	UAA	183	1.11
Leu (L)	UUG	598	1.26	Stop	UAG	158	0.96
Leu (L)	CUU	613	1.29	His (H)	CAU	494	1.44
Leu (L)	CUC	269	0.56	His (H)	CAC	192	0.56
Leu (L)	CUA	332	0.70	Gln (Q)	CAA	721	1.43
Leu (L)	CUG	267	0.56	Gln (Q)	CAG	287	0.57
Ile (I)	AUU	1071	1.40	Asn (N)	AAU	923	1.48
Ile (I)	AUC	532	0.70	Asn (N)	AAC	326	0.52
Ile (I)	AUA	692	0.90	Lys (K)	AAA	935	1.36
Met (M)	AUG	636	1.00	Lys (K)	AAG	441	0.64
Val (V)	GUU	559	1.39	Asp (D)	GAU	803	1.52
Val (V)	GUC	228	0.57	Asp (D)	GAC	257	0.48
Val (V)	GUA	568	1.41	Glu (E)	GAA	982	1.42
Val (V)	GUG	254	0.63	Glu (E)	GAG	399	0.58
Ser (S)	UCU	579	1.52	Cys (C)	UGU	270	1.27
Ser (S)	UCA	391	1.03	Cys (C)	UGC	155	0.73
Ser (S)	UCA	373	0.98	Stop	UGA	152	0.92
Ser (S)	UCG	230	0.61	Trp (W)	UGG	508	1
Pro (P)	CCU	467	1.42	Arg (R)	CGU	368	1.17
Pro (P)	CCC	253	0.77	Arg (R)	CGC	149	0.47
Pro (P)	CCA	367	1.12	Arg (R)	CGA	381	1.21
Pro (P)	CCG	228	0.69	Arg (R)	CGG	202	0.64
Thr (T)	ACU	541	1.52	Arg (R)	AGA	467	1.23
Thr (T)	ACC	313	0.88	Arg (R)	AGG	239	0.63
Thr (T)	ACA	391	1.10	Ser (S)	AGU	511	1.62
Thr (T)	ACG	181	0.51	Ser (S)	AGC	278	0.88
Ala (A)	GCU	660	1.66	Gly (G)	GGU	636	1.24
Ala (A)	GCC	273	0.69	Gly (G)	GGC	283	0.55
Ala (A)	GCA	434	1.09	Gly (G)	GGA	733	1.43
Ala (A)	GCG	225	0.57	Gly (G)	GGG	401	0.78

### Repeat Sequence and Short Sequence Repeat Analysis of the *Artemisia argyi* Chloroplast Genome

**FIGURE 3:**
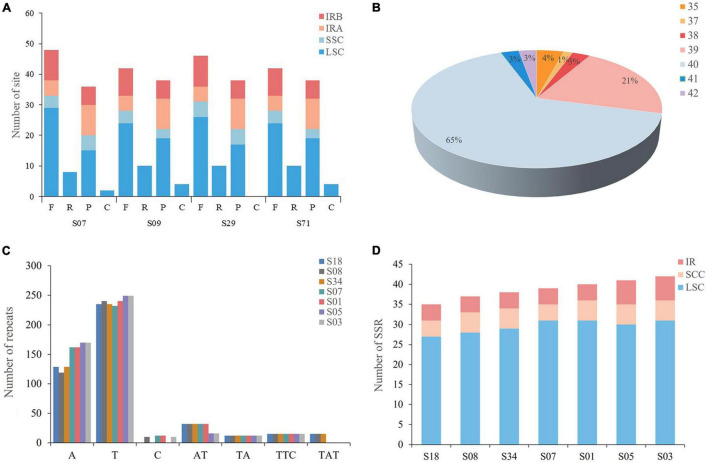
Types and distributions of repeat sequences and short sequence repeats (SSRs) in *Artemisia argyi* chloroplast (cp) genomes. **(A)** Number and position of repeat sequences in four *Artemisia argyi* cp genomes (S07, S09, S29, and S71). **(B)** Proportion of SSRs in 72 *Artemisia argyi* cp genomes. **(C)** Distribution of repeats classified by type. **(D)** The number of SSR loci in different cp genome regions.

To further analyze the characteristics of the *A. argyi* cp genome, four types of repetitive sequences were analyzed, namely, forward repeats, reverse repeats, palindromic repeats, and complementary repeats. The *A. argyi* varieties S07 (Anguo), S09 (Tangyin), S29 (Qichun), and S71 (Ninghai) were used as representative varieties, and each had 47 repeats in the cp genome. We first analyzed the length of these repeats which ranged from 19 to 48 bp. Most repeats were 20–29 bp, accounting for 80.3% of the total. The longest repeat was a palindrome sequence in the LSC that was 48 bp long. The location of these repeats was then investigated. The results showed that most forward and palindrome repeats and all reverse and complementary repeats were located in the LSC (Figure 3A).

We then determined the distribution of SSRs in the cp genomes of 72 *A. argyi* varieties. A total of 2,849 SSRs were identified. The number of SSRs in each cp genome ranged from 35 to 42, and 65.3% of *A. argyi* varieties had 40 SSRs in the cp genome (Figure 3B). To explore the characteristics of SSRs, seven varieties with different numbers of SSRs were selected for further analysis of SSR types and distributions: S18 (35 SSRs), S08 (37 SSRs), S34 (38 SSRs), S07 (39 SSRs), S01 (40 SSRs), S05 (41 SSRs), and S03 (42 SSRs). The percentages of mononucleotide, dinucleotide, and trinucleotide SSRs were 86.7, 8.6, and 4.7%, respectively (Figure 3C). With respect to distribution, a great majority of SSRs (73.2–79.5%) were located in the LSC. The IR regions had four to six SSRs; the SSC had four to five SSCs, and all trinucleotide repeats were located in the SSC. It is worth mentioning that 85.3% of the SSRs were mononucleotide A/T repeats, indicating an A/T nucleotide bias in *A. argyi* cp SSRs (Figure 3D). In addition, we developed several molecular markers based on SSR analysis of cp genomes to identify these *A. argyi* varieties, and Sanger sequencing of PCR amplification demonstrated that these SSRs were usable (Supplementary Figure 1 and Supplementary Table 2).

### Comparative Analysis of Chloroplast Genomes in 72 *Artemisia argyi* Varieties

**FIGURE 4:**
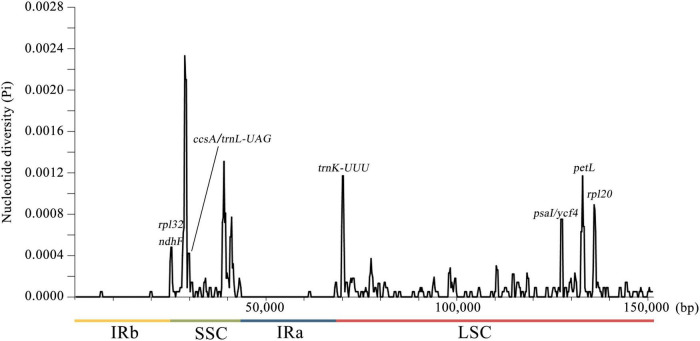
Sliding window analysis of 72 complete chloroplast (cp) genomes of *Artemisia argyi*. The *x*-axis represents the midpoint of the window and the *y*-axis represents the nucleotide diversity (Pi) of each window. The window length is 600 bp with a 200-bp step size.

**FIGURE 5:**
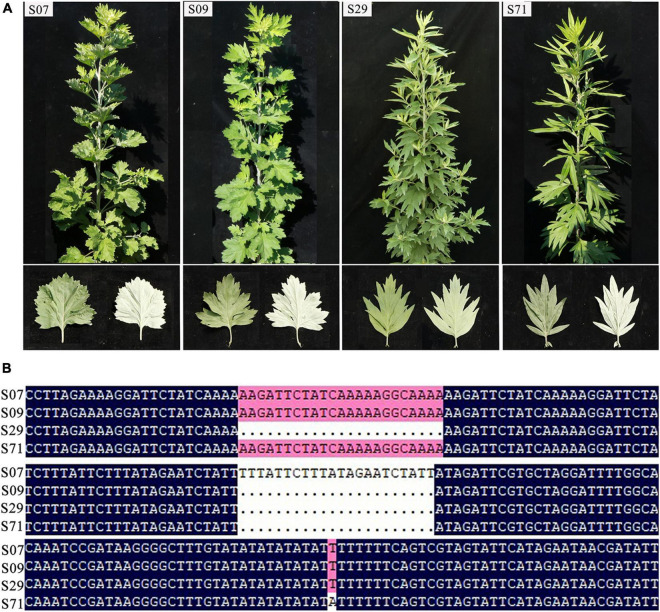
Plant morphology of S07, S09, S29, and S71 **(A)** and three polymorphic sites in chloroplast (cp) genomes of these four varieties **(B)**.

To detect the nucleotide diversity in 72 *A. argyi* cp genomes, the level of sequence variability was calculated using DnaSP v6.0. A total of 150,753 sites (excluding sites with gaps/missing data) were investigated, including 196 polymorphic sites in total (Figure 4). Among these polymorphic sites, there were 155 singleton variable sites and 41 parsimony-informative sites. The nucleotide diversity (*Pi*) values ranged from 0 to 0.00233 with an average of 0.00007, indicating that *A. argyi* cp genomes had high sequence similarity. In general, there were more variable sites in non-coding regions than in coding regions, and the variations in SC regions had greater divergence than variations in IR regions. The *Pi* values of 11 protein-coding genes (*trnK-UUU*, *petL*, *rpl20*, *psaI, ycf4*, *psbA*, *rpl32*, *ndhF*, *ccsA*, *trnL-UAG*, and *rpl32*) were higher than 0.0004; *trnK-UUU*, *petL*, and *rpl20* had the highest *Pi* values. Moreover, all the 11 genes were located in SC regions, explaining why SC regions had greater variation than IR regions. And these highly variable regions could be used to identify *A. argyi* varieties. Using the four representative *A. argyi* varieties (S07, S09, S29, and S71) as an example, 30 polymorphic sites were detected in the cp genomes, including 24 single-nucleotide polymorphisms (SNPs) and six small insertions and deletions (INDELs). Combined with the analysis of repeat sequences, we selected two repeats and one SNP for distinguishing these four *A. argyi* varieties (Figure 5). Based on these variant sites, three specific molecular markers were developed and validated through high-fidelity PCR and Sanger sequencing, and the results indicated that these markers were fit for use (Supplementary Figure 2 and Supplementary Table 3).

### Phylogenetic Analysis of 72 *Artemisia argyi* Varieties Based on Chloroplast Genomes

**FIGURE 6:**
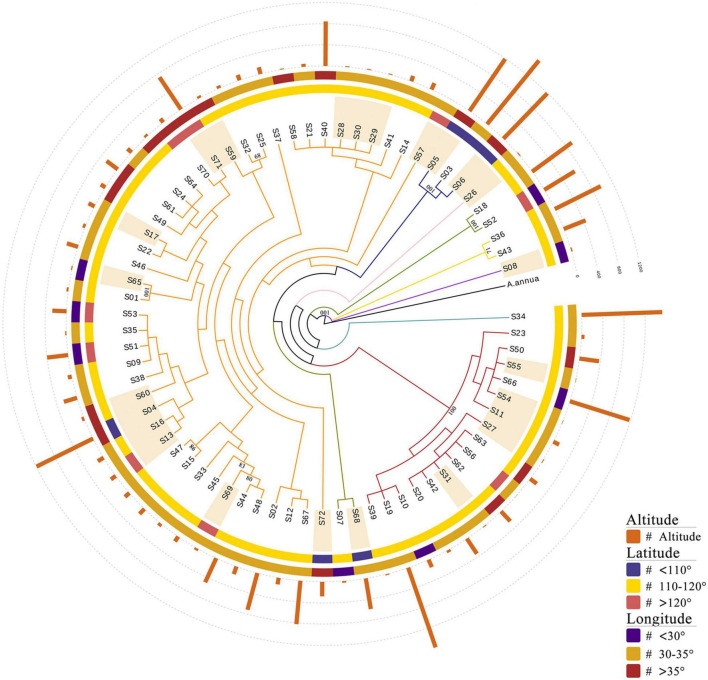
Phylogenetic analyses of *72 Artemisia argyi* varieties. The tree is constructed using the ML method based on full-length chloroplast (cp) genomes. *A. annua* is used as the outgroup. Branches are color-coded. Sample numbers marked with light orange shadow are wild germplasm resources. Bootstrap values above 70 are marked in the figure.

To investigate the genetic relationships between the 72 *A. argyi* varieties, a phylogenetic tree was constructed based on cp genomes using *A. annua* as the outgroup (Figure 6 and Supplementary Figure 4). The results showed that the varieties were divided into nine branches, with the largest branch containing 44 varieties. Because the selected germplasm resources contained both cultivated and wild varieties from wide geographical and altitudinal ranges (from 25.08 to 42.95°N, 104.9 to 129.52°E, and 2 to 1,592 m), we wondered whether the evolutionary relationships between varieties were associated with their collection locations. However, the phylogenetic analysis demonstrated that germplasm resources collected from similar longitudes or latitudes did not cluster together. Moreover, there was no obvious clustering between the cultivated and wild varieties, which may be due to the short cultivation history of *A. argyi*. Interestingly, there were 12 varieties outside the two largest branches of the phylogenetic tree, eight of which were collected at an altitude of above 500 m; this indicated that evolution of the cp genome likely occurred in the adaptive process of *A. argyi* varieties to grow at different altitudes. However, it is worth mentioning that most bootstrap values of the evolutionary tree were less than 70 due to high conservation of *A. argyi* cp genomes, indicating that the groups in this evolutionary tree were not much supported, and therefore other kinds of molecular markers would be necessary to assess phylogenetic relationships within the species *A. argyi*.

### Comparison of Boundary Regions in *Artemisia* Species

**FIGURE 7:**
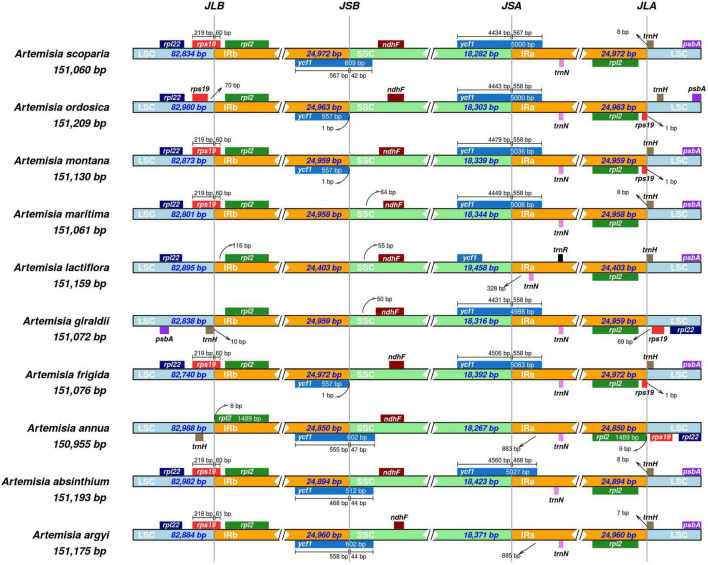
Comparison of the large single-copy (LSC), small single-copy (SSC), and inverted repeat (IR) regional boundaries of ten *Artemisia* species chloroplast (cp) genomes. JSA denotes the junction between IRa and SSC; JLA denotes the junction between IRa and LSC; JLB denotes the junction between IRb and LSC; JSB denotes the junction between IRb and SSC.

The boundary regions based on high throughput sequencing of *A. argyi* cp genomes were first validated by PCR and Sanger sequencing (Supplementary Figure 3 and Supplementary Table 4). To investigate species-specific gene loci, we compared IR/LSC and IR/SSC junction regions of the cp genomes in nine *Artemisia* species (*Artemisia absinthium* Linn., *Artemisia annua*, *Artemisia frigida* Willd., *Artemisia giraldii* Pamp., *Artemisia lactiflora* Wall ex DC., *Artemisia maritima* Linn., *Artemisia montana* Nakai., *Artemisia ordosica* Krasch and *Artemisia scoparia* Waldst. et Kit.) with *A. argyi* (S29). The results revealed that the length of IR regions ranged from 24,403 to 24,972 bp, and cp genomes in these species showed significant difference in IR/LSC and IR/SSC junction. In total, there were 10 genes around the boundaries: *rpl22, rps19, rpl2, ycf1, ndhF, trmN, rpl2, trnH, psbA*, and *trnR*. The characteristics of four boundaries, namely, IRa-SSC (JSA), IRa-LSC (JLA), IRb-LSC (JLB), and IRb-SSC (JSB), were then systematically analyzed in these species. The JLB boundary was located in *rps19* in most of the *Artemisia* cp genomes (60%). The *ycf1* gene was critical for the JSB and JSA boundaries. In four species, the JSB boundary was located in *ycf1*. In three species, the JSB boundary was only 1 bp away from *ycf1*. Furthermore, there were seven species in which the JSA boundary was in *ycf1*. For all the 10 species, the JLA boundary was between two genes; among these 10 species, the JLA boundaries were between *rpl2* and *trnH* for five species, *rpl2* and *rps19* for two, and *rps19* and *trnH* for three (Figure 7). These results demonstrated that the IR/LSC and IR/SSC junction regions varied between *Artemisia* species, likely due to an expansion or contraction of the IR region.

### Comparative Analysis of Chloroplast Genomes in *Asteraceae* Species With mVISTA

**FIGURE 8:**
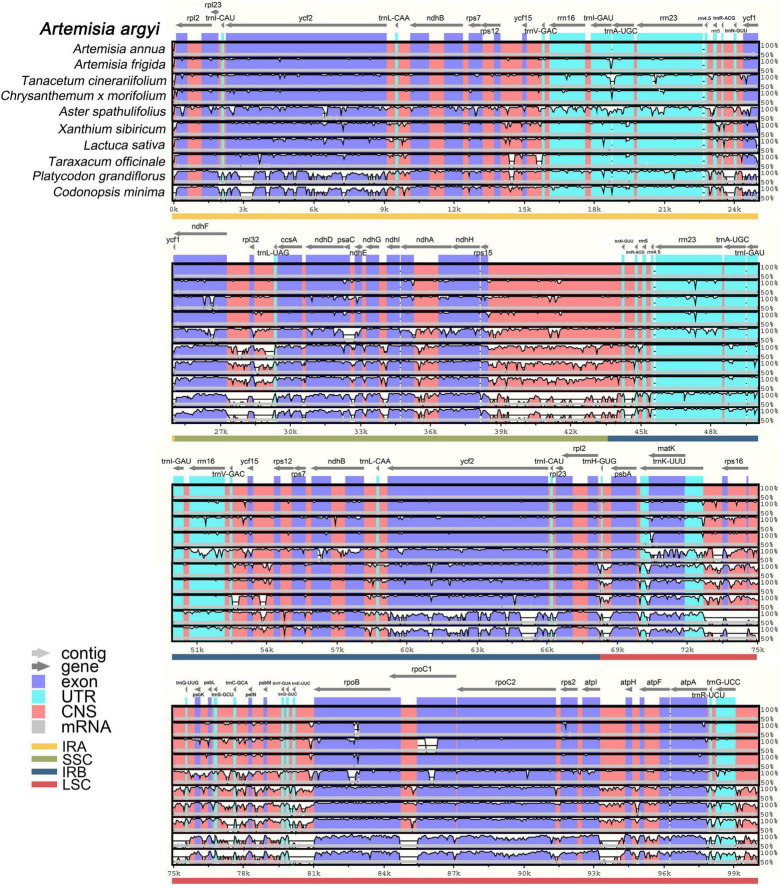
Full-length chloroplast (cp) genome alignment of *Campanulales* species with mVISTA. Using the cp genome of *Artemisia argyi* as a reference, the *x*-axis indicates the coordinates in cp genomes and the *y*-axis indicates the average percent identity, ranging from 50 to 100%. Arrows indicate gene orientation; cp genome regions are color-coded as exons, untranslated regions, conserved non-coding sequences, and mRNA.

The whole sequence identity of 10 *Campanulales* cp genomes including 8 *Asteraceae* and 2 *Campanulaceae* Juss. species was then plotted by mVISTA, using the *A. argyi* cp genome as a reference (Figure 8). Species belonging to different families had cp genomes with similar length, structure, and gene distribution, indicating that cp genomes were highly conserved in these species. The non-coding sequences were more variable than the coding sequences, and SC regions had higher levels of divergence than IR regions. Notably, intron-containing genes had higher levels of variability. In general, the species related to *A. argyi* based on classical taxonomy also had higher cp genome similarity. In addition, two species in the family *Campanulaceae* [*P. grandiflorus* (Jacq.) A. DC and *C. minima* (L.) A. Br. et Aschers.] had a larger number of variable regions, demonstrating a clear distinction from the family *Asteraceae*. The coding regions of 12 genes (*ycf2*, *trnI-GAU, trnA-UGC, ycf1, ccsA, ndhD, ndhA, trnK-UUU, rpoB, rpoC1, rpoC2*, and *trnG-UCC*) were relatively variable in non-*Anthemidinae* O. Hoffm. species in *Asteraceae*, and these genes may therefore be useful to identify *Anthemideae* Cass species. By comparison, the coding regions of four genes (*trnA-UGC, rrn23, trnK-UUU*, and *rpoB*) were relatively variable in non-*Artemisia* species of *Anthemideae* Cass, and could thus be used for identifying *Artemisia* species. Taken together, the results of the comparative cp genomic analysis provide valuable information for *Asteraceae* species identification at the molecular level.

### Phylogenetic Analysis of 67 *Asteraceae* Species

**FIGURE 9:**
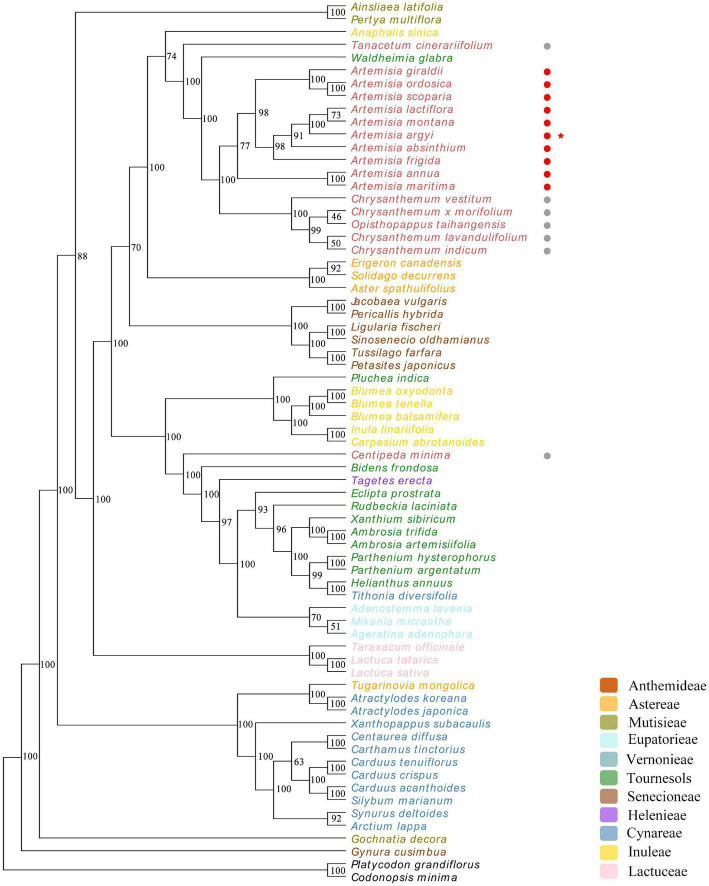
Phylogenetic tree constructed using 43 protein-coding genes common to 67 different *Asteraceae* species. *Codonopsis minima* and *Platycodon grandiflorus* from the family *Campanulaceae* are used as outgroup. The numbers above the branches represent the ML bootstrap values. Different tribes are color-coded. Non-*Artemisia* species of *Anthemideae* Cass are followed by gray circles, *Artemisia* species are followed by red circles, and *Artemisia argyi* is indicated with a red star.

To explore the evolutionary relationship of *Asteraceae* species, cp genomes of *A. argyi* and 66 other *Asteraceae* species were selected for phylogenetic analysis. Two *Campanulaceae* species (*C. minima* and *P. grandiflorus*) were used as the outgroup to detect whether *Asteraceae* and non-*Asteraceae* species can be divided into two groups based on their cp genomes. Phylogenetic trees were constructed using the ML method and seven regions of the genome: the complete cp genome, the CDS, the SSC, the LSC, the IR region, introns, and 43 protein-coding genes shared by the 67 species. The results showed that *Lactuceae* Cass could not be distinguished from *Carduoideae* Kitam based on the cp genome. Moreover, there were variations in the branches between the trees (Figure 9 and Supplementary Figure 5); the phylogenetic trees based on the IR, introns, and the 43 protein-coding genes were more consistent with the traditional classification system than those based on the full cp genome, the CDS, the SSC, or the LSC. The tree constructed from 43 common protein-coding genes had the highest reliability (89.6% of bootstrap values > 75), and the 10 *Artemisia* species included in this analysis were clustered in one branch, demonstrating a close genetic relationship. Among these 10 *Artemisia* species, *A. argyi*, *A. latiflora*, and *A. montana* formed a single group, suggesting a particularly close evolutionary relationship among the three species. Furthermore, four *Chrysanthemum* species and one *Opisthopappus* C. Shih species formed a larger branch with the 10 *Artemisia* species, suggesting that those *Chrysanthemum* and *Opisthopappus* species were closely related to *Artemisia* (Figure 9). In comparison, the other phylogenetic trees slightly differed from the traditional classification system (Supplementary Figure 5). This indicated that it was more reasonable to investigate the evolutionary relationship of *Asteraceae* species based on the shared protein-coding genes in their cp genomes.

## Discussion

Chloroplast genome provides useful information for the study of evolution. Although the *A. argyi* cp genome has previously been reported (Kang et al., 2016), there is a lack of systematic research and evolutionary analysis of *A. argyi* varieties. In this study, cp genomes of 72 *A. argyi* varieties from 47 regions in China were determined. The length of *A. argyi* cp genomes ranged from 151,115 to 151,202 bp, placing them as medium-sized cp genomes compared to other *Asteraceae* species. GC content is generally considered an important indicator of species affinity (Chen J. et al., 2021). In *A. argyi* cp genomes, the GC content was significantly higher in IR regions than that in SC regions, which was mainly due to the abundance of rRNA genes in IR regions. The cp genome of *A. argyi* contained 114 genes, similar to the cp genome of *A. annua* (Shen et al., 2017). The *A. argyi* cp genome gained *psbG* and *rpl12* but lost *trnT-GGU* in comparison to the cp genome of *A. annua*. Moreover, 13 intron-containing genes were detected in the *A. argyi* cp genome, all of which were located in the LSC. Among them, *trnK-UUU* had the largest intron, which contained *matK* (Wang Y. et al., 2021); *matK* is a well-known gene that is widely used for molecular identification and analysis of genetic relationships in medicinal plants (Hilu and Liang, 1997; Ramesh et al., 2022). Many intron-containing genes have important physiological functions; *clpP* is relevant to proteolysis (Shikanai et al., 2001) and *petB* encodes the B6 protein, the core component of the cytochrome b6f complex (Allen et al., 2011). Introns in these genes may be useful in the physiological function of *A. argyi*. Repeat sequence and SSR analyses have been widely used in studies of plant genetics, polymorphisms, and evolution. In the *A. argyi* cp genome, repeat sequences and SSRs were primarily located in the LSC region. Four *A. argyi* varieties from each of the four famous producing areas were used as representatives: S07, S09, S29, and S71. In these representative varieties, the types of repetitive sequences identified were mainly forward and palindromic repeats. The cp genomes of most *A. argyi* varieties contained ∼40 SSRs including mononucleotide, dinucleotide, and trinucleotide repeats. Most of the SSRs identified were mononucleotide A/T repeats, consistent with the cp genomes in most angiosperms (Hu et al., 2016).

Polymorphic sites and evolutionary relationships were here investigated in 72 *A. argyi* cp genomes. Overall, 196 polymorphic sites were detected. IR regions were found to be more conserved than SC regions. These polymorphic sites provide valuable information for the identification of *A. argyi* varieties at the molecular level. Previous studies indicated that *A. argyi* germplasm resources are rich in genetic diversity (Chen C. J. et al., 2021). Based on intraspecific evolutionary analysis of *A. argyi*, there was no obvious clustering of *A. argyi* varieties from similar longitudes, latitudes, or cultivation histories. However, germplasm resources collected from higher altitudes were more likely to cluster away from other germplasm resources based on their cp genomes. This may be the result of the following two factors: First, *A. argyi* has a short history of artificial domestication, so the cultivation and domestication have not yet had a remarkable impact on the conserved cp genomes. Second, the results suggest that altitude may have a greater impact on *A. argyi* evolution than longitude and latitude.

The morphological characteristics of *Artemisia* species are similar. By comparing cp genome sequences, the genetic differences between species can be clearly observed at the molecular level. Taken together, the cp genomes of ten selected *Artemisia* species were relatively conserved in the LSC/IR boundaries and more variable in the SSC/IR boundaries. *Ycf1* and *rps19* are considered pseudogenes that do not encode proteins (Vanin, 1985). There are a total of eight genes around the boundaries of these four regions in the *A. argyi* cp genome. *Rps19* spans the LSC/IRb boundary and *ycf1* spans the IRb/SSC boundary; this was also seen in *A. scoparia* and *A. absinthium*. However, *rps19* was not detected in *Aster spathulifolius*, which is similar to some *Asteraceae* species including *Centaurea diffusa* Lam., *Chrysanthemum indicum* L., *Chrysanthemum × morifolium* Ramat. *Jacobaea vulgaris* Gaertn. and *Lactuca sativa* Linn. in the family *Asteraceae* (Liang et al., 2018). In addition to *A. lactiflora*, other *Artemisia* species contain these two pseudogenes, which have been duplicated in *A. frigida*, *A. montana*, and *A. ordosica*. Taken together, the copy numbers and positions of *ycf1* and *rps19* in *Artemisia* species are quite different, consistent with *Arecaceae* Bercht. and J. Presl (de Santana Lopes et al., 2018) and herbaceous bamboos (Wang et al., 2018). In contrast, *rpl2*, *ndhF*, and *trnN* are relatively conserved in the IR boundary of *Artemisia*. The mVISTA analysis of 11 *Asteraceae* cp genomes showed that variable regions were primarily located in SC regions and that the coding region was more conserved than the non-coding region, which is consistent with the results of the polymorphism analysis in the 72 *A. argyi* cp genomes. Relevant research indicates that this phenomenon is observed in most higher plants, such as the families *Styracaceae* DC. and Spreng. (Cai et al., 2021) and *Rosaceae* Juss. (Xue et al., 2019). The higher conservation observed in the IR region may be due to copy correction caused by gene transformation between the two IR regions (Zhang et al., 2016). In previous studies, several most informative coding regions (*ycf1*, *rps16*, *ccsA*, *rbcL*, *ndhA*, *matK*, *clpP*, and *accD*) were usually used for developing specific barcodes in the *Asteraceae* family, which is different from the identification of *A. argyi* varieties (Curci et al., 2015).

*Asteraceae* contains a wide variety of species that are extensively geographically distributed, which makes classification extremely complex at both the morphological and molecular levels. The cp genome is widely used to study the evolutionary relationships between species due to its high conservation and slow evolution rate. To explore the phylogenetic relationships between *Asteraceae* species and to clarify the phylogenetic position of *A. argyi*, 7 phylogenetic trees were constructed using different regions of the cp genomes of *A. argyi* and 66 other *Asteraceae* species. The resulting seven phylogenetic trees were roughly similar to one another and to the traditional taxonomic classification. The tree built using the 43 protein-coding genes shared by the 67 species was closest to the traditional classification system, a finding that is consistent with related studies in other species (Shen et al., 2017; Xue et al., 2019). We therefore conducted our main analysis using the tree constructed with the 43 common protein-coding genes. The results showed that *A. argyi* is closely related to other *Artemisia* species, and that *Artemisia* species are closely related to other *Anthemideae* Cass species. *Anthemideae* Cass species have a closer relationship with some *Astereae* Cass species and a slightly more distant relationship with some *Senecioneae* Cass species. It is worth mentioning that the evolutionary relationships of the 67 *Asteraceae* species based on cp genomes were not completely consistent with traditional taxonomic classifications. We speculate that this may be for three reasons: (1) those species underwent genetic variation in the process of evolution; (2) the evolutionary speed of different species is diverse, and for some rapidly evolving species, the phylogenetic relationships of these species may not be accurately reflected based on the relatively conserved cp genome (Moore et al., 2010); and (3) cp DNA is parthenogenetic, so the cp genomes can only reflect the evolutionary processes of maternal or paternal lines (Feng et al., 2020).

## Conclusion

In this study, we sequenced the cp genomes of 72 *A. argyi* varieties from 47 regions in China, and the characteristics of *A. argyi* cp genomes were systematically analyzed. A comparative analysis of repeat sequences and SSRs in *A. argyi* cp genomes provided abundant variation sites for developing molecular markers of variety identification. Despite the high conservation of *A. argyi* cp genomes, 196 polymorphic sites are still detected. And the difference between the IR boundaries of ten *Artemisia* species provides useful information for identifying different *Artemisia* species. Moreover, the phylogenetic relationship of 67 *Asteraceae* species shows that *A. argyi* is closely related to *A. lactiflora* and *A. Montana*. Based on the relationship between *Anthemideae Cass* and other tribes, it appeared that *A. Cass* species had a closer relationship with some *Astereae* Cass species, and a slightly more distant relationship with some *Senecioneae* Cass species. These results provide key novel insights for *A. argyi* variety identification and understanding of the evolutionary history of the family *Asteraceae*.

## Data Availability Statement

The datasets presented in this study can be found in online repositories. The raw data of 72 cp genome sequencing have been deposited to NCBI under the accession number PRJNA821831 and the assembled 72 cp genomes were deposited in the CNGB Sequence Archive (CNSA) of the China National Gene Bank Database (https://db.cngb.org/) under the accession number CNP0002996.

## Author Contributions

DLi and TZ designed the research. CC, TZ, YM, DLu, JL, ZW, and ML performed experiments. CC and TZ analyzed the data and wrote the manuscript. All authors have read and approved the final manuscript.

## Conflict of Interest

The authors declare that the research was conducted in the absence of any commercial or financial relationships that could be construed as a potential conflict of interest.

## Publisher’s Note

All claims expressed in this article are solely those of the authors and do not necessarily represent those of their affiliated organizations, or those of the publisher, the editors and the reviewers. Any product that may be evaluated in this article, or claim that may be made by its manufacturer, is not guaranteed or endorsed by the publisher.

## Footnotes

1
https://www.ldmap.net/index.html

2
https://www.ncbi.nlm.nih.gov/

3
http://www.biomarker.com.cn/

4
https://github.com/ndierckx/NOVOPlasty

5
http://www.geneious.com

6
http://lowelab.ucsc.edu/tRNAscan-SE/

7
https://irscope.shinyapps.io/Chloroplot/

8
http://bibiserv.techfak.uni-bielefeld.de/reputer/

9
https://webblast.ipk-gatersleben.de/misa/

10
https://irscope.shinyapps.io/irapp/

11
http://genome.lbl.gov/vista/mvista/submit.shtml

## References

[B1] Allen J. F. de Paula W. B. Puthiyaveetil S. Nield J. (2011). A structural phylogenetic map for chloroplast photosynthesis. *Trends Plant Sci*. 16 645–655. 10.1016/j.tplants.2011.10.004 22093371

[B2] Amiryousefi A. Hyvonen J. Poczai P. (2018). IRscope: an online program to visualize the junction sites of chloroplast genomes. *Bioinformatics* 34 3030–3031. 10.1093/bioinformatics/bty220 29659705

[B3] Bansod S. Chilvery S. Saifi M. A. Das T. J. Tag H. Godugu C. (2021). Borneol protects against cerulein-induced oxidative stress and inflammation in acute pancreatitis mice model. *Environ*. *Toxicol*. 36 530–539. 10.1002/tox.23058 33166053

[B4] Cai X. L. Landis J. B. Wang H. X. Wang J. H. Zhu Z. X. Wang H. F. (2021). Plastome structure and phylogenetic relationships of *Styracaceae* (Ericales). *BMC*. *Ecol*. *Evol*. 21:103. 10.1186/s12862-021-01827-4 34049486 PMC8161964

[B5] Cardini F. Weixin H. (1998). Moxibustion for correction of breech presentation: a randomized controlled trial. *JAMA*. 280 1580–1584. 10.1001/jama.280.18.1580 9820259

[B6] Chen C. J. Luo D. D. Miao Y. H. Guo L. P. Liu D. H. (2021). Diversity of *Artemisia argyi* germplasm resources based on agronomic and leaf phenotypic traits. *Zhongguo Zhong Yao Za Zhi* 46 2773–2782. 10.19540/j.cnki.cjcmm.20210125.101 34296575

[B7] Chen J. Zang Y. Shang S. Liang S. Zhu M. Wang Y. (2021). Comparative chloroplast genomes of *Zosteraceae* species provide adaptive evolution insights into seagrass. *Front*. *Plant Sci*. 12:741152. 10.3389/fpls.2021.741152 34630493 PMC8495015

[B8] Cui Y. Gao X. Wang J. Shang Z. Zhang Z. Zhou Z. (2021). Full-length transcriptome analysis reveals candidate genes involved in terpenoid biosynthesis in *Artemisia argyi*. *Front. Genet.* 12:659962. 10.3389/fgene.2021.659962 34239538 PMC8258318

[B9] Curci P. L. Paola D. D. Danzi D. Vendramin G. G. Sonnante G. (2015). Complete chloroplast genome of the multifunctional crop globe artichoke and comparison with other Asteraceae. *PLoS One* 10:e0120589. 10.1371/journal.pone.0120589 25774672 PMC4361619

[B10] Curci P. L. Paola D. D. Sonnante G. (2016). Development of chloroplast genomic resources for Cynara. *Mol. Ecol. Resour.* 16 562–573. 10.1111/1755-0998.12457 26354522

[B11] de Santana Lopes A. Gomes Pacheco T. Nimz T. do Nascimento Vieira L. Guerra M. P. Nodari R. O. (2018). The complete plastome of macaw palm [*Acrocomia aculeata* (Jacq.) Lodd. ex Mart.] and extensive molecular analyses of the evolution of plastid genes in *Arecaceae*. *Planta* 247 1011–1030. 10.1007/s00425-018-2841-x 29340796

[B12] Dierckxsens N. Mardulyn P. Smits G. (2017). NOVOPlasty: de novo assembly of organelle genomes from whole genome data. *Nucleic Acids Res.* 45:e18. 10.1093/nar/gkw955 28204566 PMC5389512

[B13] Doyle J. J. Doyle J. L. Doyle J. A. Doyle F. J. (1987). A rapid DNA isolation procedure for small amounts of fresh leaf tissue. *Phytochem. Bull.* 19 11–13.

[B14] Du Z. Lu K. Zhang K. He Y. Wang H. Chai G. (2021). The chloroplast genome of *Amygdalus* L. (Rosaceae) reveals the phylogenetic relationship and divergence time. *BMC Genomics* 22:645. 10.1186/s12864-021-07968-6 34493218 PMC8425060

[B15] Favier A. Gans P. Boeri Erba E. Signor L. Muthukumar S. S. Pfannschmidt T. (2021). The plastid-encoded RNA polymerase-associated protein PAP9 is a superoxide dismutase with unusual structural features. *Front. Plant Sci.* 12:668897. 10.3389/fpls.2021.668897 34276730 PMC8278866

[B16] Feng S. Zheng K. Jiao K. Cai Y. Chen C. Mao Y. (2020). Complete chloroplast genomes of four *Physalis* species (Solanaceae): lights into genome structure, comparative analysis, and phylogenetic relationships. *BMC Plant Biol.* 20:242. 10.1186/s12870-020-02429-w 32466748 PMC7254759

[B17] Gao X. Zhang X. Meng H. Li J. Zhang D. Liu C. (2018). Comparative chloroplast genomes of Paris Sect. Marmorata: insights into repeat regions and evolutionary implications. *BMC Genomics* 19:878. 10.1186/s12864-018-5281-x 30598104 PMC6311911

[B18] Hilu K. Liang H. (1997). The *matK* gene: sequence variation and application in plant systematics. *Am. J. Bot.* 84 830. 21708635

[B19] Hu Y. Woeste K. E. Zhao P. (2016). Completion of the chloroplast genomes of five Chinese Juglans and their contribution to chloroplast phylogeny. *Front. Plant Sci.* 7:1955. 10.3389/fpls.2016.01955 28111577 PMC5216037

[B20] Kang S. H. Kim K. Lee J. H. Ahn B. O. Won S. Y. Sohn S. H. (2016). The complete chloroplast genome sequence of medicinal plant, *Artemisia argyi*. *Mitochondrial DNA B Resour.* 1 257–258. 10.1080/23802359.2016.1159926 33473468 PMC7799501

[B21] Katoh K. Standley D. M. (2013). MAFFT multiple sequence alignment software version 7: improvements in performance and usability. *Mol. Biol. Evol.* 30 772–780. 10.1093/molbev/mst010 23329690 PMC3603318

[B22] Kawabe A. Nukii H. Furihata H. Y. (2018). Exploring the history of chloroplast capture in *Arabis* using whole chloroplast genome sequencing. *Int. J. Mol. Sci.* 19:602. 10.3390/ijms19020602 29463014 PMC5855824

[B23] Kearse M. Moir R. Wilson A. Stones-Havas S. Cheung M. Sturrock S. (2012). Geneious Basic: an integrated and extendable desktop software platform for the organization and analysis of sequence data. *Bioinformatics* 28 1647–1649. 10.1093/bioinformatics/bts199 22543367 PMC3371832

[B24] Kim Y. J. Kim S. Jung Y. Jung E. Kwon H. J. Kim J. (2018). Eupatilin rescues ciliary transition zone defects to ameliorate ciliopathy-related phenotypes. *J. Clin. Invest.* 128 3642–3648. 10.1172/JCI99232 30035750 PMC6063470

[B25] Kurtz S. Schleiermacher C. (1999). REPuter: fast computation of maximal repeats in complete genomes. *Bioinformatics* 15 426–427. 10.1093/bioinformatics/15.5.426 10366664

[B26] Lee J. H. Lee J. W. Sung J. S. Bang K. H. Moon S. G. (2009). Molecular authentication of 21 Korean *Artemisia* species (Compositae) by polymerase chain reaction-restriction fragment length polymorphism based on trnL-F region of chloroplast DNA. *Biol. Pharm. Bull.* 32 1912–1916. 10.1248/bpb.32.1912 19881307

[B27] Lee J. H. Lee Y. J. Lee J. Y. Park Y. M. (2017). Topical application of *Eupatilin Ameliorates* atopic dermatitis-like skin lesions in NC/Nga mice. *Ann. Dermatol.* 29 61–68. 10.5021/ad.2017.29.1.61 28223748 PMC5318529

[B28] Li C. Cai C. Tao Y. Sun Z. Jiang M. Chen L. (2021). Variation and evolution of the whole chloroplast genomes of *Fragaria* spp. (Rosaceae). *Front. Plant Sci.* 12:754209. 10.3389/fpls.2021.754209 34721483 PMC8551639

[B29] Liang F. Wen X. Gao H. Ying Z. (2018). Analysis of chloroplast genomes features of *Asteraceae* species. *Genomics Appl. Biol.* 37 5437–5447. 10.13417/j.gab.037.005437

[B30] Librado P. Rozas J. (2009). DnaSP v5: a software for comprehensive analysis of DNA polymorphism data. *Bioinformatics* 25 1451–1452. 10.1093/bioinformatics/btp187 19346325

[B31] Liu M. Zhu J. Wu S. Wang C. Guo X. Wu J. (2018). De novo assembly and analysis of the *Artemisia argyi* transcriptome and identification of genes involved in terpenoid biosynthesis. *Sci. Rep.* 8:5824. 10.1038/s41598-018-24201-9 29643397 PMC5895812

[B32] Lowe T. M. Chan P. P. (2016). tRNAscan-SE On-line: integrating search and context for analysis of transfer RNA genes. *Nucleic Acids Res.* 44 54–57. 10.1093/nar/gkw413 27174935 PMC4987944

[B33] Martins A. Rodrigues L. B. Cesario F. de Oliveira M. R. C. Tintino C. D. M. Castro F. F. E. (2017). Anti-edematogenic and anti-inflammatory activity of the essential oil from *Croton rhamnifolioides* leaves and its major constituent 1,8-cineole (eucalyptol). *Biomed. Pharmacother.* 96 384–395. 10.1016/j.biopha.2017.10.005 29031196

[B34] Mei Q. X. Chen X. L. Xiang L. Liu Y. Su Y. Y. Gao Y. Q. (2016). DNA barcode for identifying folium *Artemisiae Argyi* from counterfeits. *Biol. Pharm. Bull.* 39 1531–1537. 10.1248/bpb.b16-00336 27582332

[B35] Moore M. J. Soltis P. S. Bell C. D. Burleigh J. G. Soltis D. E. (2010). Phylogenetic analysis of 83 plastid genes further resolves the early diversification of eudicots. *Proc. Natl. Acad. Sci. U.S.A.* 107 4623–4628. 10.1073/pnas.0907801107 20176954 PMC2842043

[B36] Mulyaningsih S. Sporer F. Zimmermann S. Reichling J. Wink M. (2010). Synergistic properties of the terpenoids aromadendrene and 1,8-cineole from the essential oil of Eucalyptus globulus against antibiotic-susceptible and antibiotic-resistant pathogens. *Phytomedicine* 17 1061–1066. 10.1016/j.phymed.2010.06.018 20727725

[B37] Qiao J. W. Cai M. X. Yan G. X. Wang N. Li F. Chen B. Y. (2016). High-throughput multiplex cpDNA resequencing clarifies the genetic diversity and genetic relationships among *Brassica napus*, *Brassica rapa* and *Brassica oleracea*. *Plant Biotechnol. J.* 14 409–418. 10.1111/pbi.12395 26031705 PMC11388923

[B38] Ramesh G. A. Mathew D. John K. J. Ravisankar V. (2022). Chloroplast gene *matK* holds the barcodes for identification of *Momordica* (Cucurbitaceae) species from Indian subcontinent. *Hortic. Plant J.* 8 89–98. 10.1016/j.hpj.2021.04.001

[B39] Shen X. Wu M. Liao B. Liu Z. Bai R. Xiao S. (2017). Complete chloroplast fenome sequence and phylogenetic analysis of the medicinal plant *Artemisia annua*. *Molecules* 22 1330. 10.3390/molecules22081330 28800082 PMC6152406

[B40] Shikanai T. Shimizu K. Ueda K. Nishimura Y. Kuroiwa T. Hashimoto T. (2001). The chloroplast *clpP* gene, encoding a proteolytic subunit of ATP-dependent protease, is indispensable for chloroplast development in tobacco. *Plant Cell Physiol.* 42 264–273. 10.1093/pcp/pce031 11266577

[B41] Shinozaki K. Ohme M. Tanaka M. Wakasugi T. Hayashida N. Matsubayashi T. (1986). The complete nucleotide sequence of the tobacco chloroplast genome: its gene organization and expression. *EMBO J.* 5 2043–2049. 10.1002/j.1460-2075.1986.tb04464.x 16453699 PMC1167080

[B42] Song Y. Chen Y. Lv J. Xu J. Zhu S. Li M. (2017). Development of chloroplast genomic resources for *Oryza* species discrimination. *Front. Plant Sci.* 8:1854. 10.3389/fpls.2017.01854 29118779 PMC5661024

[B43] Stamatakis A. (2014). RAxML version 8: a tool for phylogenetic analysis and post-analysis of large phylogenies. *Bioinformatics* 30, 1312–1313. 10.1093/bioinformatics/btu033 24451623 PMC3998144

[B44] Thiel T. Michalek W. Varshney R. K. Graner A. (2003). Exploiting EST databases for the development and characterization of gene-derived SSR-markers in barley (*Hordeum vulgare* L.). *Theor. Appl. Genet.* 106 411–422. 10.1007/s00122-002-1031-0 12589540

[B45] Vanin E. F. (1985). Processed pseudogenes: characteristics and evolution. *Annu. Rev. Genet.* 19 253–272. 10.1146/annurev.ge.19.120185.001345 3909943

[B46] Wang M. Wang X. Sun J. Wang Y. Ge Y. Dong W. (2021). Phylogenomic and evolutionary dynamics of inverted repeats across *Angelica* plastomes. *BMC Plant Biol.* 21:26. 10.1186/s12870-020-02801-w 33413122 PMC7792290

[B47] Wang W. Chen S. Zhang X. (2018). Whole-genome comparison reveals divergent IR borders and mutation hotspots in chloroplast genomes of *Herbaceous Bamboos* (Bambusoideae: Olyreae). *Molecules* 23:1537. 10.3390/molecules23071537 29949900 PMC6099781

[B48] Wang Y. Wang S. Liu Y. Yuan Q. Sun J. Guo L. (2021). Chloroplast genome variation and phylogenetic relationships of *Atractylodes* species. *BMC Genomics* 22:103. 10.1186/s12864-021-07394-8 33541261 PMC7863269

[B49] Xue S. Shi T. Luo W. Ni X. Iqbal S. Ni Z. (2019). Comparative analysis of the complete chloroplast genome among *Prunus mume*, *P. armeniaca*, and *P. salicina*. *Hortic. Res.* 6:89. 10.1038/s41438-019-0171-1 31666958 PMC6804877

[B50] Zhang Y. Du L. Liu A. Chen J. Wu L. Hu W. (2016). The complete chloroplast genome sequences of five *Epimedium* species: lights into phylogenetic and taxonomic analyses. *Front. Plant Sci.* 7:306. 10.3389/fpls.2016.00306 27014326 PMC4791396

