# Supplementary material for: Sequence Characteristics and Phylogenetic Analysis of the Artemisia argyi Chloroplast Genome
Source: Front Plant Sci. 2022 Jun 20;13:906725. doi: 10.3389/fpls.2022.906725 (PMC9252292; doi:10.3389/fpls.2022.906725)
Supplement: Supplementary file 1 [file Data_Sheet_1.pdf]

Supplementary Material

Figure. S1

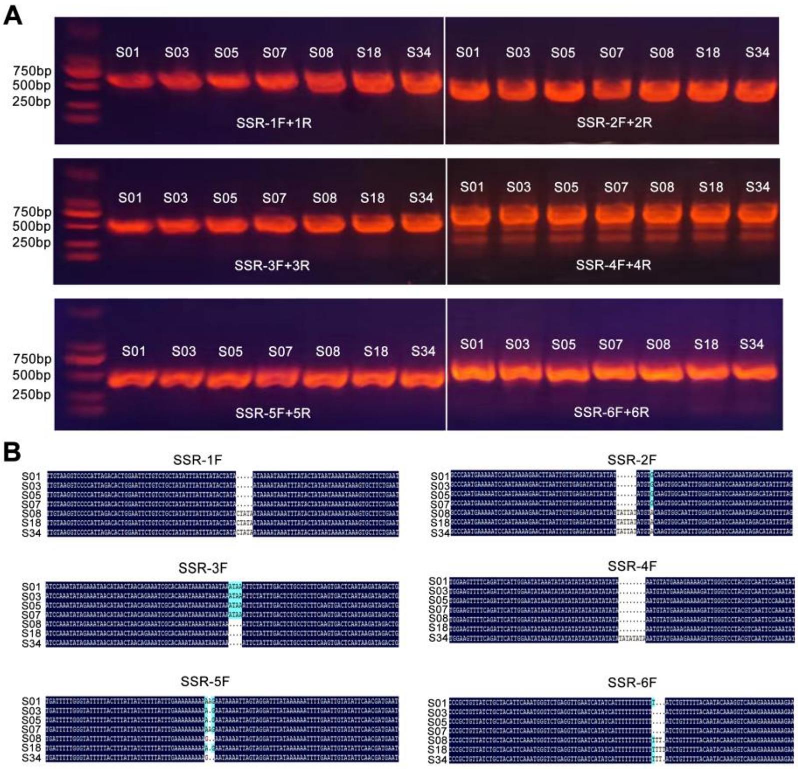

Fig. S1 Identification of seven *Artemisia argyi* varieties (S01, S03, S05, S07, S08, S18 and S34) based on SSRs.

(A) The primers used for the identification of these seven *Artemisia argyi* varieties are listed in Table S2. The corresponding PCR products are amplified using the genomic DNA of S01, S03, S05, S07, S08, and S71, respectively. **(B)** The results of Sanger sequencing, which are consistent with the SSR analysis based on *Artemisia argyi* cp genomes.

Figure. S2

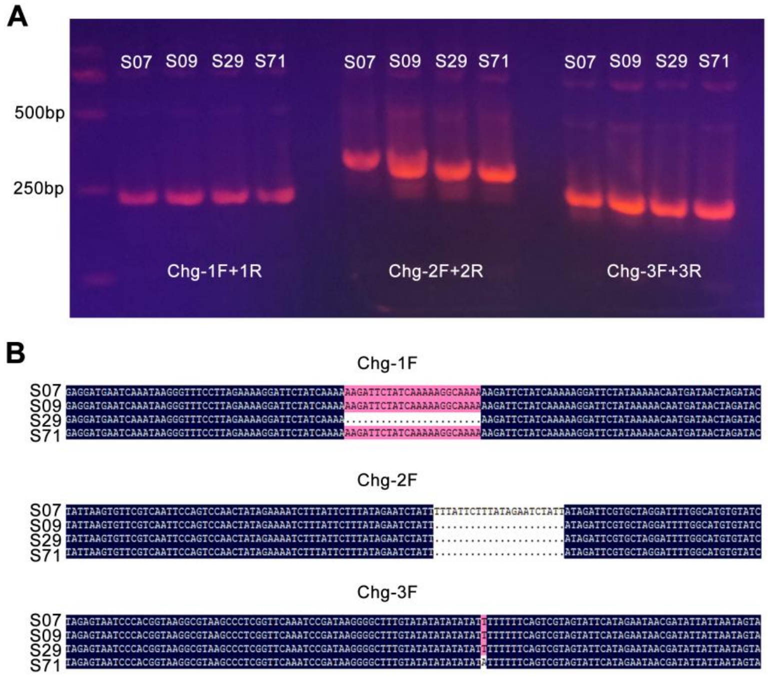

Fig. S2 Identification of four representative *Artemisia argyi* varieties (S07, S09, S29, and S71).

(A) Three pairs of primers (Chg-1F+1R, Chg-2F+2R, and Chg-3F+3R) are used to identify these four representative *Artemisia argyi* varieties. The corresponding PCR products are amplified using the genomic DNA of S07, S09, S29, and S71, respectively. **(B)** Sanger sequencing of PCR products. And the results are consistent with corresponding assembled *Artemisia argyi* cp genomes from high throughput sequencing.

Figure. S3

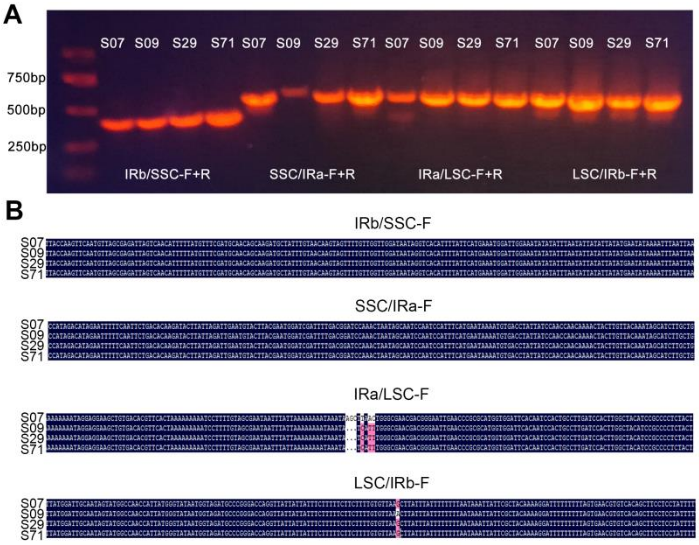

Fig. S3 The validation of IR/SC boundaries in *Artemisia argyi* cp genomes.

(A) Four pairs of primers (IRb/SSC-F+R, SSC/IRa-F+R, IRa/LSC-F+R, and LSC/IRb-F+R) are used to validate IR/SC boundaries. The corresponding PCR products are amplified using the genomic DNA of S07, S09, S29, and S71, respectively. **(B)** Sanger sequencing of PCR products. The results are consistent with corresponding assembled *Artemisia argyi* cp genomes from high throughput sequencing.

Figure. S4

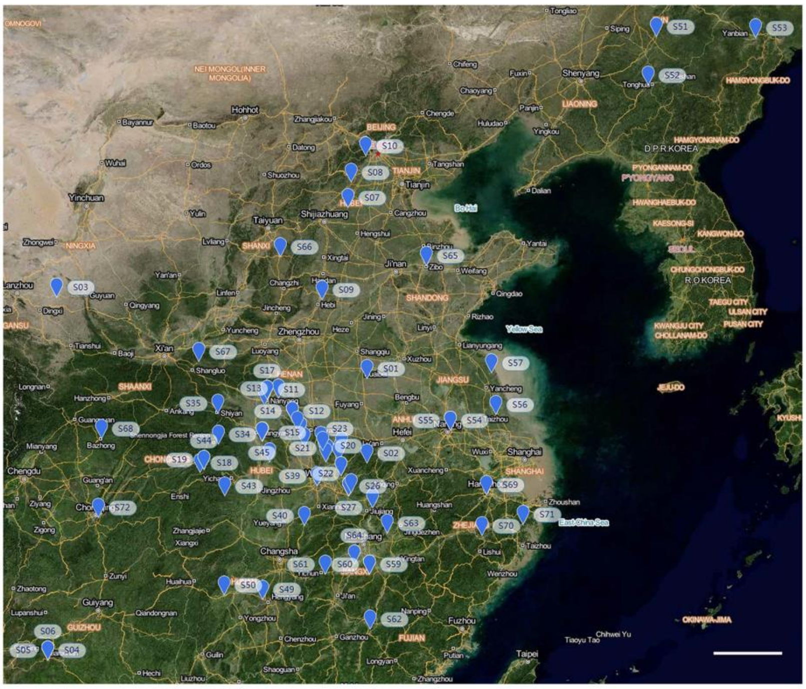

Fig. S4 The distribution of *Artemisia argyi* varieties.

Scale bar = 20 km.

Figure. S5

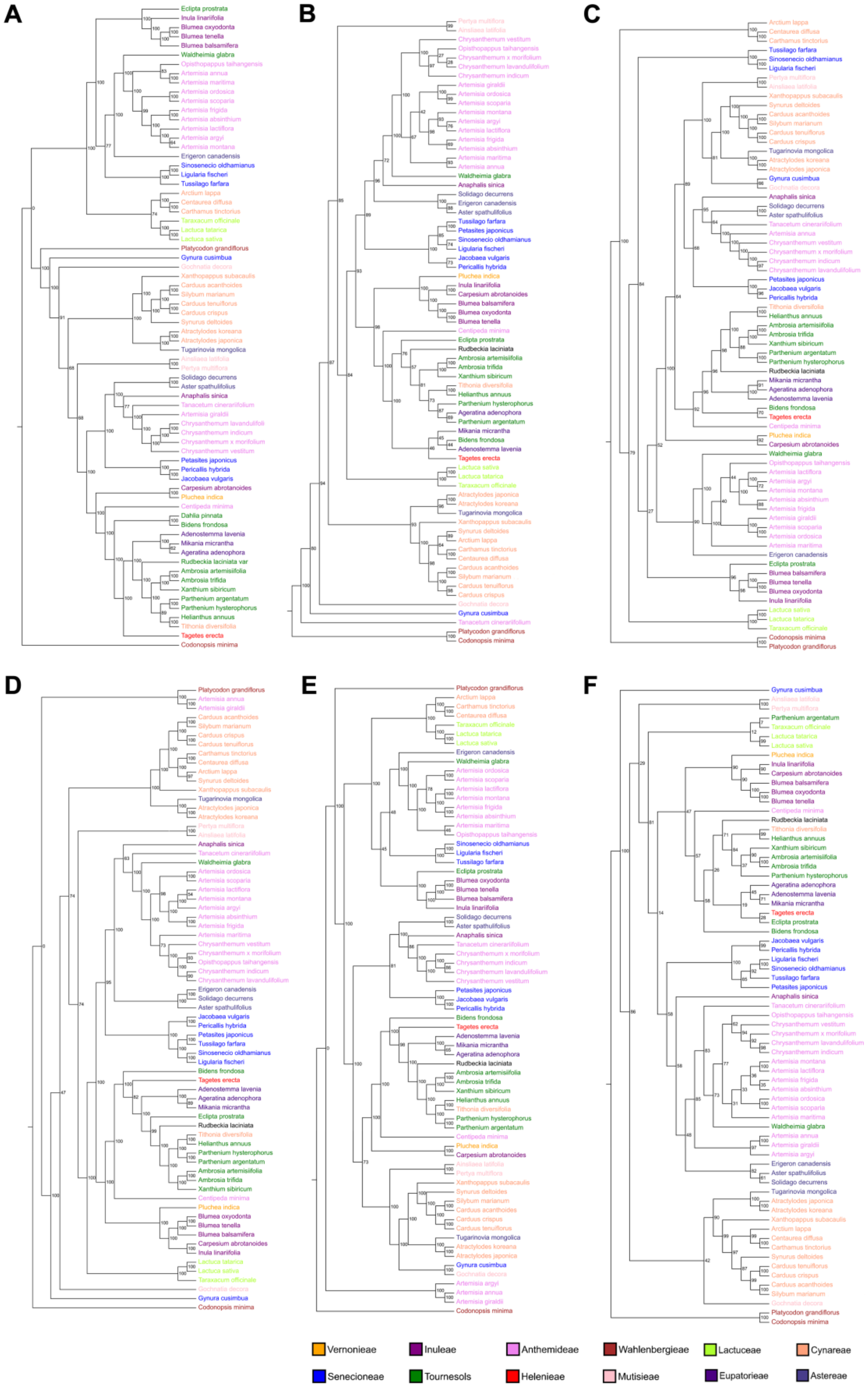

Fig.S5 Phylogenetic trees of 67 *Asteraceae* species based on their cp genomes by ML.

(A) Phylogenetic tree based on the full-length cp genomes; **(B)** Phylogenetic tree based on IR regions; **(C)** Phylogenetic tree based on SSC regions; **(D)** Phylogenetic tree based on LSC regions; **(E)** Phylogenetic tree based on CDSs; **(F)** Phylogenetic tree based on introns.

Table S1 Description of *Artemisia argyi* accessions in this study.

	Collecting locality	Longitude (˚E)	Latitude (˚N)	Altitude (m)	Type	Sample number	Full length of cp genome (bp)
Anhui	Qiaocheng, Bozhou	115.99	33.62	37	Wild	S01	151177
	Huoshan, Lu‘an	115.99	31.13	719	Wild	S02	151180
Gansu	Xihe, Longnan	105.20	35.95	1553	Wild	S03	151177
Guizhou	Pengzha, Xingyi	104.90	25.08	1193	Cultivated	S04	151178
	Pengzha, Xingyi	104.90	25.08	1193	Cultivated	S05	151176
	Pengzha, Xingyi	104.90	25.08	1193	Cultivated	S06	151177
Hebei	Anguo, Baoding	115.33	38.42	30	Wild	S07	151202
	Xushui, Baoding	115.46	39.12	24	Wild	S08	151148
Henan	Tangyin, Anyang	114.44	35.87	73	Wild	S09	151178
	Fangcheng, Nanyang	115.95	39.83	153	Wild	S10	151152
	Sheqi,Nanyang	112.52	33.00	121	Cultivated	S11	151151
	Sheqi,Nanyang	113.40	32.37	125	Wild	S12	151172
	Sheqi,Nanyang	112.93	33.05	112	Cultivated	S13	151176
	Tongbai, Nanyang	113.40	32.37	132	Wild	S14	151174
	Tongbai, Nanyang	113.40	32.37	132	Wild	S15	151179
	Wuolong, Nanyang	112.45	32.84	127	Cultivated	S16	151178
	Wuolong, Nanyang	112.52	33.00	127	Cultivated	S17	151196
Hubei	Badong, Enshi	110.20	30.84	931	Wild	S18	151179
	Badong, Enshi	110.34	30.94	1592	Wild	S19	151177
	Hong'an, Huanggang	114.51	31.35	48	Wild	S20	151173
	Hong'an, Huanggang	114.61	31.27	55	Wild	S21	151151
	Hong'an, Huanggang	114.56	31.16	50	Wild	S22	151199
	Macheng, Huanggang	115.05	31.29	56	Wild	S23	151173
	Macheng, Huanggang	115.11	31.48	102	Wild	S24	151152
	Qichun, Huanggang	115.02	31.18	106	Wild	S25	151178
	Qichun, Huanggang	115.36	30.14	20	Cultivated	S26	151115
	Qichun, Huanggang	115.36	30.14	16	Cultivated	S27	151153
	Qichun, Huanggang	115.36	30.14	16	Cultivated	S28	151175
	Qichun, Huanggang	115.36	30.14	16	Cultivated	S29	151175
	Qichun, Huanggang	115.36	30.14	16	Cultivated	S30	151175
	Qichun, Huanggang	115.44	30.23	16	Cultivated	S31	151178
	Tuanfeng, Huanggang	115.10	30.75	53	Wild	S32	151175
	Tuanfeng, Huanggang	115.10	30.75	58	Wild	S33	151151
	Shuichiya, Shenlongjia	110.84	31.68	1526	Wild	S34	151167
	Maojian, Shiyan	110.82	32.60	22	Wild	S35	151178
	Guangshui, Suizhou	113.83	31.62	969	Wild	S36	151178
	Guangshui, Suizhou	113.70	31.94	130	Wild	S37	151151
	Suixian, Suizhou	113.59	32.09	259	Wild	S38	151157
	Hongshan, Wuhan	114.26	30.45	20	Wild	S39	151183

Table S1 Description of *Artemisia argyi* accessions in this study (continued).

	Tongcheng, Xianning	113.82	29.25	843	Wild	S40	151175
	Dawu, Xiaogan	114.39	31.65	106	Wild	S41	151175
	Dawu, Xiaogan	114.47	31.51	343	Wild	S42	151178
	Wufeng, Yichang	111.06	30.14	445	Wild	S43	151195
	Wufeng, Yichang	110.34	30.94	501	Wild	S44	151152
	Baokang, Xiangyang	112.36	31.78	110	Wild	S45	151133
	Baokang, Xiangyang	112.37	31.79	220	Wild	S46	151177
	Baokang, Xiangyang	112.36	31.78	110	Wild	S47	151133
	Baokang, Xiangyang	112.61	31.16	152	Wild	S48	151173
Hunan	Hengyang, Hengyang	112.38	26.98	102	Wild	S49	151178
	Longhui, Shaoyang	111.04	27.12	373	Wild	S50	151151
Jilin	Panshi, Jilin	126.07	42.95	401	Wild	S51	151178
	Tonghua, Tonghua	125.77	41.69	676	Wild	S52	151167
	Yanji, Yanbian	129.52	42.91	269	Wild	S53	151178
Jiangsu	Qixia, Nanjing	118.91	32.10	9	Cultivated	S54	151151
	Qixia, Nanjing	118.91	32.10	9	Cultivated	S55	151151
	Hai'an, Nantong	120.48	32.57	4	Wild	S56	151151
	Sheyang, Yancheng	120.33	33.77	2	Wild	S57	151179
Jiangxi	Jiangzhou, Chaisang	116.19	29.79	16	Wild	S58	151175
	Chongren, Fuzhou	116.08	27.76	44	Cultivated	S59	151177
	Chongren, Fuzhou	116.08	27.76	46	Cultivated	S60	151180
	Quannan, Ganzhou	114.54	27.75	263	Wild	S61	151178
	Rentian, Ruijin	116.10	26.05	255	Wild	S62	151151
	Poyang, Shangrao	116.71	29.01	154	Wild	S63	151151
	Zhangshu, Yichun	115.55	28.06	45	Wild	S64	151178
Shandong	Hengtai, Zibo	118.06	36.82	20	Cultivated	S65	151179
Shaanxi	Yushe, Jinzhong	112.98	37.08	1182	Wild	S66	151151
Shanxi	Luonan, Shangluo	110.15	34.10	823	Wild	S67	151181
Sichuan	Bazhou, Bazhong	106.78	31.86	587	Cultivated	S68	151133
Zhejiang	Bingjiang, Hangzhou	120.16	30.18	42	Cultivated	S69	151174
	Yongkang, Jinhua	120.00	28.93	87	Wild	S70	151178
	Ninghai, Ningbo	121.43	29.29	730	Cultivated	S71	151178
Chongqing	Nan’an	106.67	29.50	275	Cultivated	S72	151175

Table S2 Molecular markers used for the identification of S01, S03, S05, S07, S08, S18 and S34.

Primer name	Primer sequence
SSR-1F	TTTGACTGCGCTTCCACTATA
SSR-1R	TGAGAAAGGAGAATAGGAACGA
SSR-2F	CTTTTCCCCATCGACTTTTAA
SSR-2R	TTTGACGTAGTTTAGCCGATTC
SSR-3F	AATAGTCATTGGTTCAGTCGGTA
SSR-3R	GAAAACTAAATTGCATCCCTAAA
SSR-4F	TTTGACTCTGGACCATTCATTC
SSR-4R	TGCTGAAATCAAGGGGCTATA
SSR-5F	AGTACTCAAGCGTTTCGGATT
SSR-5R	TTTCCATAAAGGAGCCGAAT
SSR-6F	AGTTCCTTTTGGATTTCCTTCT
SSR-6R	AGTTACAGTAAGACCCGCAGAA

Table S3 Molecular markers used for the identification of S07, S09, S29 and S71.

Primer name	Primer sequence
Chg-1F	TCTTACTTGATATTAGAGGTC
Chg-1R	TGGATATCTTTTCTATACTATGT
Chg-2F	CGAATAGCGGGACCAAAA
Chg-2R	TCCTCCGATTCTCAAAGGAG
Chg-3F	ATAGTACAGTGACCCGTTCAGG
Chg-3R	TTCGTGATAACTATAACTAAACTCATT

Table S4 Primers used for the verification of IR/SC boundaries in *Artemisia argyi* cp genomes.

Primer name	Primer sequence
IRb/SSC-F	TGCGTAATCTCAGCATTCAAT
IRb/SSC-R	GAATTCCAAATGGAGTTGGTC
SSC/IRa-F	TTTTCCTGATCCCCGTACATA
SSC/IRa-R	GCTCTACCGTATCTTTTGTTTCAT
IRa/LSC-F	CTTGGGGTTATCCTGCACTT
IRa/LSC-R	GTTCGATTCGCGTGTTTTCT
LSC/IRb-F	CGACGAGACCTATTATCGCTT
LSC/IRb-R	GGTAAGCGCCCTGTAGTAAGAG

Table S5 Molecular markers used for the identification of *Asteraceae* species.

Primer name	Primer sequence
rrn23-1F	AACACAGGTCTCCGCAAAGTCGT
rrn23-1R	TGCACGCCTCCGTTACCTTTTGG
rrn23-2F	GAAACCGACACAGGTGGGTAGGT
rrn23-2R	CACTTGGCTACCCAGCGTTTACC
ccsA-1F	ATGATATTTTCAACTTTAGAGCA
ccsA-1R	CACAAAGAAGAGCCGCATAGCTC
ccsA-2F	TTTCTTTCATGGAGTTTCTCC
ccsA-2R	CAAATTATAAGAAAGCCTAGAG
ndhA-1F	ATCAATTCTTTTTCTAGATTGGA
ndhA-1R	CATAACTAATTGATTGAGCAGC
ndhA-2F	AGAGAATCTTCTTCCATCTAGAG
ndhA-2R	CAACCCCCCAAGTAAAGAACTGT
ndhD-1F	CATAAAGGAAATAGGGTAATTA
ndhD-1R	ACCGATACCCAGAACTCCCAT
ndhD-2F	ACAAATAATCTATGCAGCTTCA
ndhD-2R	AGTTAATATCATTCCAATTGCCAT
ropB-1F	CTCTAATGACTTCTCTGGGAG
ropB-1R	ATCTCGTATTGGAAAAGTAGC
ropB-2F	CTAGTCACTATGGGCGTATTTG
ropB-2R	TCCCATGGCATATAAGCTACTA
ropC1-F	TCCCATGGCATATAAGCTACTA
ropC1-R	CAAAAGATTCATATGAGAAAA
ropC2-1F	GTGAATATTTGCGACAAGAAAT
ropC2-1R	ATGTTCTCGCGCGAATCTCAGCA
ropC2-2F	TCAAGGAACTATCCATACGTTGT
ropC2-2R	TCTATATGCCTATTATGTATCTG
ycf1-F	ATGATTTTGAAATCTTTTCTACT
ycf1-R	GGTAAAATGAAATGATTGAATA
ycf2-1F	TCAATCCAGAGATCATTTTGAT
ycf2-1R	TCTTCTGCTCAGAAACGAAATG
ycf2-2F	AGATCGATTCTTTGGGATCCTT
ycf2-2R	GAAATAGGATGAATTGAGACGGT

Table S6 Information of *Asteraceae* cp genomes obtained from NCBI.

No.	Species	ID
1	*Artemisia argyi*	NC030785.1
2	*Adenostemma lavenia*	MW583043.1
3	*Ageratina adenophora*	NC015621.1
4	*Ainsliaea latifolia*	NC056135.1
5	*Ambrosia artemisiifolia*	MG019037.1
6	*Ambrosia trifida*	NC036810.2
7	*Anaphalis sinica*	NC034648.1
8	*Arctium lappa*	MH375874.1
9	*Artemisia absinthium*	MK188885.1
10	*Artemisia annua*	MF623173.1
11	*Artemisia frigida*	NC020607.1
12	*Artemisia giraldii*	OK128342.1
13	*Artemisia lactiflora*	MZ151340.1
14	*Artemisia maritima*	MK532038.1
15	*Artemisia montana*	NC025910.1
16	*Artemisia ordosica*	NC046571.1
17	*Artemisia scoparia*	NC045286.1
18	*Aster spathulifolius*	NC027434.1
19	*Atractylodes japonica*	NC056949.1
20	*Atractylodes koreana*	NC056987.1
21	*Bidens frondosa*	NC050965.1
22	*Blumea balsamifera*	BK013127.1
23	*Blumea oxyodonta*	BK013128.1
24	*Blumea tenella*	BK013129.1
25	*Carduus acanthoides*	NC053725.1
26	*Carduus crispus*	NC053726.1
27	*Carduus tenuiflorus*	NC053727.1
28	*Carpesium abrotanoides*	MT663326.1
29	*Carthamus tinctorius*	KX822074.1
30	*Centaurea diffusa*	NC024286.1
31	*Centipeda minima*	MZ169540.1
32	*Chrysanthemum indicum*	MH165290.1
33	*Chrysanthemum lavandulifolium*	NC057202.1
34	*Chrysanthemum vestitum*	NC057203.1
35	*Chrysanthemum x morifolium*	NC020092.1
36	*Codonopsis minima*	NC036311.1
37	*Dahlia pinnata*	MW589541.1
38	*Eclipta prostrata*	NC030773.1
39	*Erigeron canadensis*	MT806101.1
40	*Gochnatia decora*	NC057205.1
41	*Gynura cusimbua*	NC056914.1

Table S6 Information of Asteraceae cp genomes obtained from NCBI (continued).

42	*Helianthus annuus*	NC007977.1
43	*Inula linariifolia*	MN954653.1
44	*Jacobaea vulgaris*	NC015543.1
45	*Lactuca sativa*	AP007232.1
46	*Lactuca tatarica*	NC058613.1
47	*Ligularia fischeri*	NC039352.1
48	*Mikania micrantha*	NC031833.1
49	*Opisthopappus taihangensis*	NC042787.1
50	*Parthenium argentatum*	NC013553.1
51	*Parthenium hysterophorus*	MT576959.1
52	*Pericallis hybrida*	NC031898.1
53	*Pertya multiflora*	NC054328.1
54	*Petasites japonicus*	NC056924.1
55	*Platycodon grandiflorus*	MZ202358.1
56	*Pluchea indica*	NC038194.1
57	*Rudbeckia laciniata*	MN518844.1
58	*Silybum marianum*	NC028027.1
59	*Sinosenecio oldhamianus*	NC057622.1
60	*Solidago decurrens*	NC053705.1
61	*Synurus deltoides*	NC046830.1
62	*Tagetes erecta*	MN203535.1
63	*Tanacetum cinerariifolium*	NC047309.1
64	*Taraxacum officinale*	NC030772.1
65	*Tithonia diversifolia*	MT576958.1
66	*Tugarinovia mongolica*	NC057258.1
67	*Tussilago farfara*	MW760850.1
68	*Waldheimia glabra*	NC058010.1
69	*Xanthium sibiricum*	MH473582.1
70	*Xanthopappus subacaulis*	MT643189.1
